# Characterization of a Novel Myrosinase with High Activity from Marine Bacterium *Shewanella baltica* Myr-37

**DOI:** 10.3390/ijms231911258

**Published:** 2022-09-24

**Authors:** Qinwen Ye, Yaowei Fang, Mengjiao Li, Haoyu Mi, Shu Liu, Guang Yang, Jing Lu, Yaling Zhao, Qitong Liu, Wei Zhang, Xiaoyue Hou

**Affiliations:** 1 Jiangsu Key Laboratory of Marine Bioresources and Environment, Jiangsu Ocean University, Lianyungang 222005, China; 2 Co-Innovation Center of Jiangsu Marine Bio-Industry Technology, Jiangsu Ocean University, Lianyungang 222005, China; 3 Jiangsu Marine Resources Development Research Institute, Jiangsu Ocean University, Lianyungang 222005, China; 4 College of Food Science and Engineering, Jiangsu Ocean University, Lianyungang 222005, China; 5 College of Life Sciences, Nankai University, Tianjin 300071, China

**Keywords:** microbial enzyme, myrosinase, sinigrin, glucoraphanin, sulforaphane

## Abstract

Myrosinase can hydrolyze glucosinolates to generate isothiocyanates, which have cancer prevention and anti-cancer properties. The main sources of myrosinase are cruciferous plants. To further improve the efficiency of isothiocyanates preparation, it is necessary to explore novel sources of myrosinases. In this study, we described a bacterium, *Shewanella baltica* Myr-37, isolated from marine mud, capable of producing a novel myrosinase (Smyr37) with a molecular weight of 100 kDa. The crude enzyme of Smyr37 showed the highest activity at 50 °C and pH 8.0. The sinigrin- and glucoraphanin-hydrolyzing activities of Smyr37 were 6.95 and 5.87 U/mg, respectively. Moreover, when the reaction temperature was 40 °C and pH was 7.0, the crude enzyme of Smyr37 could efficiently degrade glucoraphanin into sulforaphane within 25 min with a yield of 0.57 mg/mL. The corresponding conversion efficiency of sulforaphane from glucoraphanin was 89%. In summary, *S. baltica* Myr-37 myrosinase Smyr37, a novel myrosinase, can be used in the preparation of isothiocyanates.

## 1. Introduction

Myrosinase, a β-thioglucoside glucohydrolase, allows isothiocyanates, nitriles, and thiocyanates to be produced from glucosinolates, an essential class of sulfur-containing secondary metabolite mainly found in cruciferous plants [1,2]. These hydrolysates not only help the family *Brassicaceae* to combat with crucifer specialists, but also assist the crucifer specialists in identifying, locating, and laying eggs on the cruciferous plants [3,4]. Glucosinolates have little biological activity, but can be hydrolyzed by myrosinase to produce active metabolites such as isothiocyanates [5]. Isothiocyanates have many beneficial biological effects, such as preventing carcinogenesis and cardiovascular disease, relieving inflammation, enhancing immune and antioxidant functions, and neuroprotection [6,7,8]. Furthermore, isothiocyanates also exhibit significant antibacterial activity and are used in agriculture and the food industry [9].

**Figure 1:**
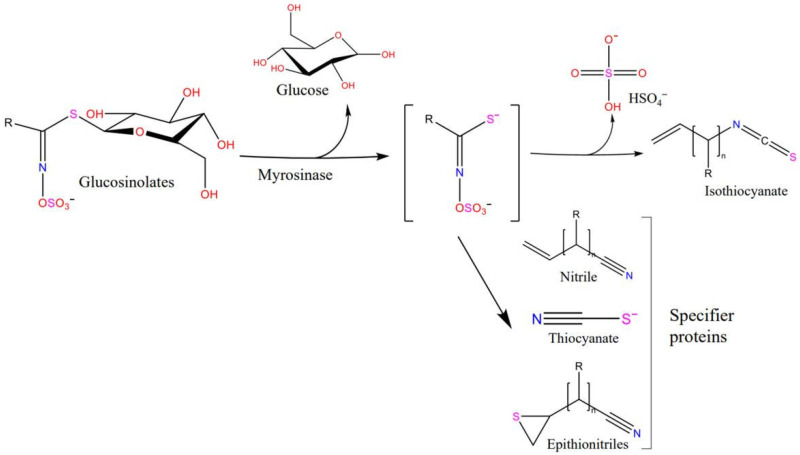
Generalized scheme of the hydrolysis of glucosinolates by myrosinase.

Based on the R groups in the side chain, glucosinolates are classified as aliphatic, aromatic, or indole. The primary process of glucosinolate degradation is as follows: glucosinolate is hydrolyzed by myrosinase to produce glucose and an unstable intermediate, which is further hydrolyzed to isothiocyanate, nitrile, or sulfocyanate based on the difference in R groups (Figure 1). For example, glucoraphanin, an aliphatic glucosinolate, can be hydrolyzed by myrosinase to produce sulforaphane, sulforaphane nitrile, goitrin, iberin, and sulforaphane was the most hydrolysate [10]. However, owing to the instability of indole isothiocyanate, the hydrolysis products of 3-indole methyl glucosinolate are mainly indole 3-carbinol and indole-3-acetonitrile [11]. In addition, the metabolites of glucosinolates are also affected by the enzymatic reaction conditions, such as pH and temperature. 

Although the non-isothiocyanate metabolites of glucosinolates also show some antibacterial activity, the potent anticancer activity of isothiocyanates has attracted increasing attention from researchers [12,13]. The effects of sulforaphane (4-methylsulfinylbutyl isothiocyanate), one of the most active isothiocyanates, on various tumor types have been investigated in several studies [14,15,16]. Plant tissue endogenous myrosinases are often used to prepare sulforaphane. However, epithiospecifier proteins located in cruciferous plant tissues can participate in the rearrangement of the intermediate glucosinolate aglycones and promote the formation of undesirable nitrile by-products, resulting in the reduction of the sulforaphane yield [17]. In addition, the content and activity of myrosinase vary in different plant tissues, which seriously affects the efficiency of sulforaphane preparation. Studies have shown that the exogenous addition of highly active myrosinase to the raw materials (without epithiospecifier protein) improves the conversion rate of raw materials to sulforaphane [18,19]. Therefore, exploring new sources of myrosinases with high activity and outstanding properties has become a research focus.

Myrosinase was first found in *Brassica* plants, such as cabbage, broccoli, and mustard greens [20]. Myrosinase is also present in some insects that host plants that contain myrosinase, such as *Brevicoryne brasicae* and *Lipaphis erysimi* [21,22]. In addition, to accelerate the industrialization of myrosinases, bacterial myrosinases are being increasingly investigated. At present, only a few studies have been conducted on bacterial myrosinases. *Citrobacter* myrosinase (Cmyr) was the first bacterial myrosinase to be discovered, with no sequence homology to plant or aphid myrosinases [23]. Myrosinases from *Leclercia adecarboxylata* can produce allyl-isothiocyanate from sinigrin [24]. The gene encoding *Rahnella inusitata* myrosinase was heterologously expressed in *Escherichia coli* BL21 (DE3), and the purified myrosinase showed excellent catalytic efficiency for the preparation of sulforaphane [14]. Although some studies have shown that human gut bacteria can convert glucosinolates to sulforaphane, the corresponding bacteria have not yet been isolated [25]. 

To accelerate the convenient application of isothiocyanates, it is necessary to explore more sources of myrosinase. In this study, we isolated a marine bacterium from the Yellow Sea, named *Shewanella baltica* Myr-37, capable of producing a novel myrosinase (Smyr37, 110 kDa). We analyzed the enzymatic characteristics of Smyr37 and purified Smyr37. In addition, the optimum conditions of Smyr37 for sulforaphane production from glucoraphanin were investigated, demonstrating its potential application as a biotechnological tool for producing high value-added isothiocyanates.

## 2. Results

### 2.1. Isolation and Identification of Myrosinase-Producing Bacteria

**Figure 2:**
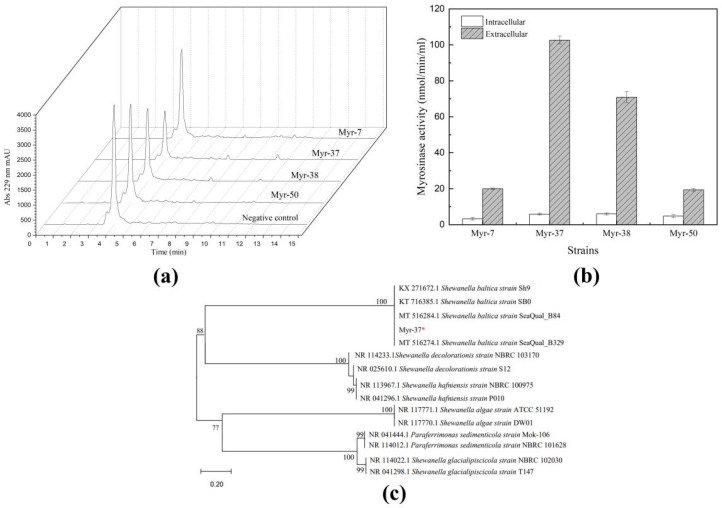
*Shewanella baltica* Myr-37 is capable of producing myrosinase. (**a**) High-performance liquid chromatography analysis at 229 nm of the sinigrin content in the fermentation broth. (**b**) Myrosinase activity of different strains. (**c**) Phylogenetic analysis of Myr-37 strain constructed using the neighbor-joining method based on 16S rRNA gene sequences. *: the strain isolated in our study.

Over 65 strains were isolated from the marine mud samples. To find bacteria capable of degrading sinigrin, high-performance liquid chromatography (HPLC) was used to analyze the sinigrin consumption. HPLC analysis showed that the sinigrin content decreased in the fermentation broth of the following four strains: Myr-7, Myr-37, Myr-38, and Myr-50 (Figure 2a). Then, the myrosinase activity of the sinigrin-degrading strains were analyzed separately by detecting the glucose production. As shown in Figure 2b, among the four strains, Myr-37 exhibited the highest extracellular myrosinase activity (102 nmol/min/mL). Moreover, the myrosinase activity of Myr-37 did not significantly increase under the condition of supplementation with sinigrin in the LB medium. Therefore, Myr-37 was selected for further investigation.

To identify the strain Myr-37, we performed 16S rRNA gene sequencing analysis, which showed 99.93% identity with the sequence of the *Shewanella baltica* (Figure 2c). This result confirmed that Myr-37 could be assigned to the species *Shewanella baltica.* The 16S rRNA gene sequence of Myr-37 had been submitted to GenBank with the accession number OL440972.1. The myrosinase produced by *Shewanella baltica* Myr-37 was named Smyr37 in this study.

### 2.2. Characterization of Shewanella baltica Myr-37 Myrosinase

**Figure 3:**
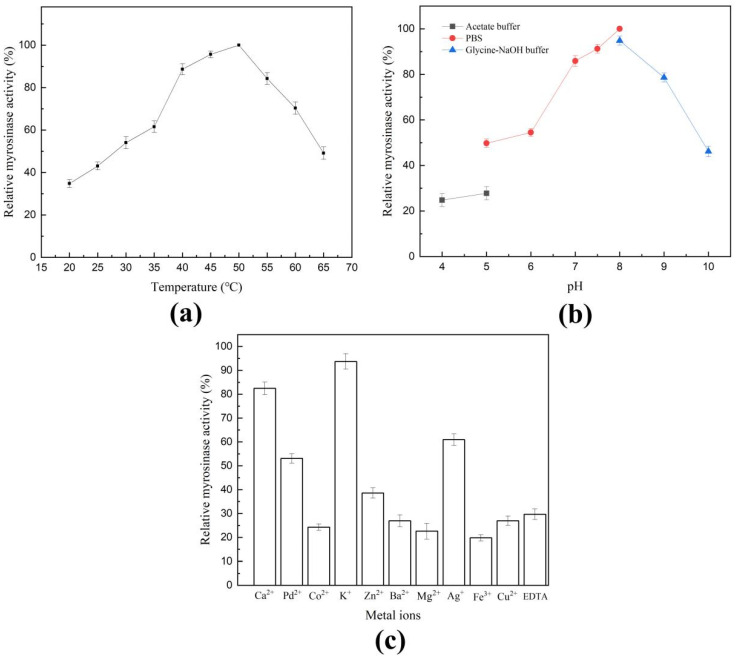
Characterization of *Shewanella baltica* Myr-37 myrosinase. Effects of temperature (**a**), pH (**b**), and metal ions (**c**) on the activity of Smyr37. The crude enzyme of Smyr37 (50 μg) and the corresponding buffer containing sinigrin (2 mg/mL) were used to perform the enzyme reactions.

Myrosinase activity could not be detected in the cell lysate, indicating that *S. baltica* Myr-37 myrosinase Smyr37 is an extracellular enzyme. To determine the optimal conditions for myrosinase activity, the effects of temperature, pH, and metal ions on myrosinase activity were preliminarily studied using crude enzyme (the concentrated liquid). As indicated in Figure 3a, Smyr37 was active over a wide temperature range from 35 °C to 60 °C, with an optimal activity at 50 °C. When the reaction temperature was 50 °C, the optimum pH for Smyr37 was 8.0 (Figure 3b). In addition, Smyr37 activity was inhibited by all the ions studied at 1 mM, except K^+^, which had a weaker effect (Figure 3c). These results showed that Smyr37 was sensitive to ions, but tolerated a relatively wide range of temperatures.

### 2.3. Purification of Shewanella baltica Myr-37 Myrosinase

**Figure 4:**
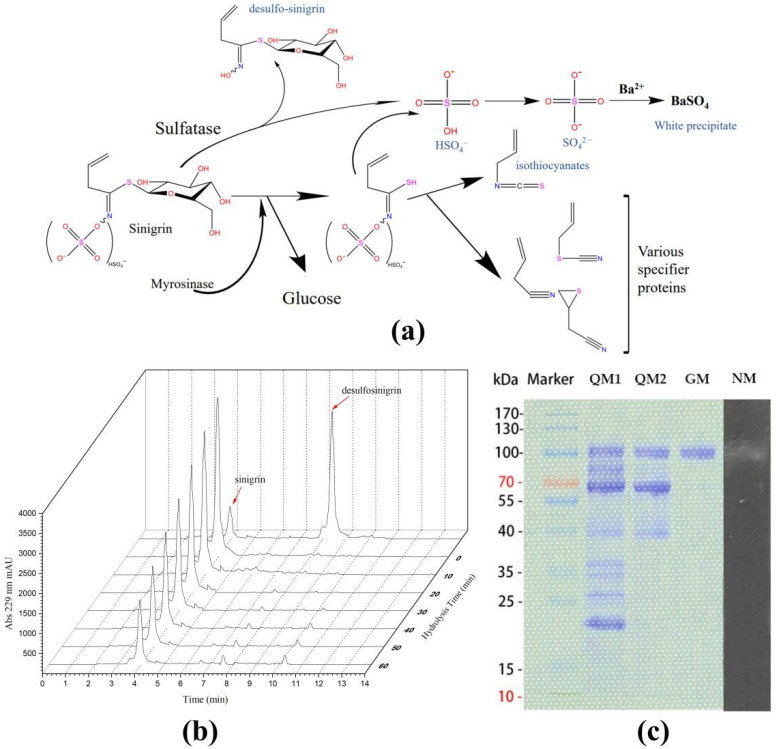
The molecular weight of *Shewanella baltica* Myr-37 myrosinase is approximately 100 kDa. (**a**) Main pathways of sinigrin metabolism. (**b**) High-performance liquid chromatography analysis of desulfo-sinigrin during the enzymatic hydrolysis. The crude enzyme (50 μg) was added to the mixture of sinigrin (2 mg/mL) in PBS to make a final volume of 1 mL. (**c**) Results of the SDS-PAGE analysis for the purification of Smyr37 and the Native-PAGE analysis of Smyr37. Lanes: QM1, ion exchange first run; QM2, ion exchange second run; GM: gel filtration; NM: Native-PAGE analysis.

Sinigrin can be catalyzed by myrosinase (or sulfatase) to release SO_4_^2−^, which reacts with Ba^2+^ to form the white precipitate (Figure 4a). The position of the white bands of BaSO_4_ in the lane of a gel indicates the molecular weight of myrosinase (or sulfatase). The crude enzyme of Smyr37 was used to hydrolyze sinigrin. The absence of desulfo-sinigrin during the enzymatic hydrolysis indicated that *S. baltica* Myr-37 might not produce sulfatase, or that sulfatase was not secreted outside the cell (Figure 4b). We used the Native-PAGE gel with Ba^2+^ to determine the molecular weight of myrosinase in the fermentation solution. As shown in Figure 4c, the appearance of a single white band of BaSO_4_ in the Native-PAGE gel indicated that the approximate molecular weight of Smyr37 was 100 kDa. Smyr37 was then purified using ion exchange chromatography and gel filtration. Finally, we obtained a pure single band with a molecular weight of approximately 100 kDa on the SDS-PAGE gel (Figure 4c). The purification summary of Smyr37 is shown in Table 1; we obtained 15.9 μg of myrosinase from a 500 mL fermentation broth.

### 2.4. Catalytic Activity of Shewanella baltica Myr-37 Myrosinase against Glucoraphanin

**Figure 5:**
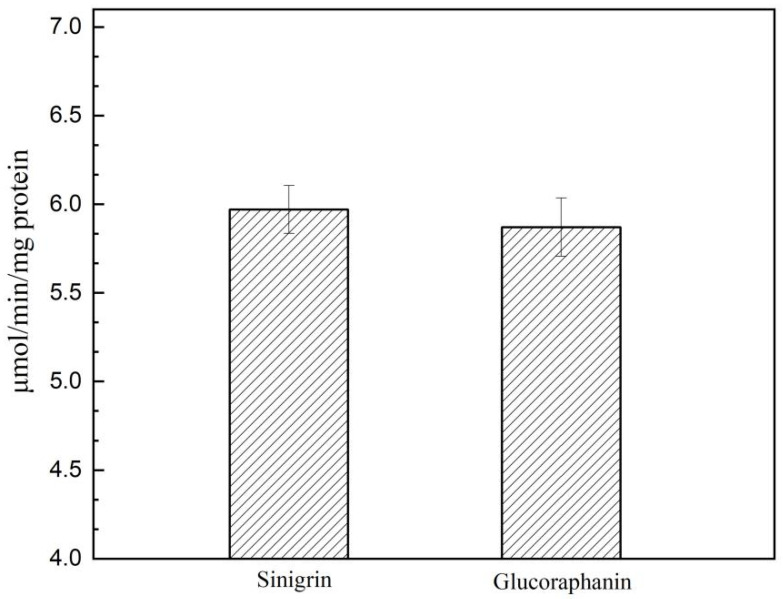
Hydrolysis of sinigrin and glucoraphanin by *Shewanella baltica* Myr-37 myrosinase toward sinigrin compared with glucoraphanin. Purified Smyr37 (2 μg) was added to the mixture of sinigrin/glucoraphanin (5 mM) in PBS to make a final volume of 1 mL.

The side chain of glucosinolates affects the catalytic activity of myrosinase. Glucoraphanin is an aliphatic glucosinolate and its degradation product, sulforaphane, has strong anti-cancer activity. To investigate whether Smyr37 is active against glucoraphanin, the purified Smyr37 was used for the hydrolysis of glucoraphanin. With a hydrolysis time of 30 min, the sinigrin- and the glucoraphanin-hydrolyzing activities of Smyr37 were 6.95 μmol/min/(mg protein) and 5.87 μmol/min/(mg protein), respectively (Figure 5). These results indicated that Smyr37 was active toward glucoraphanin, but showed a better affinity for sinigrin than glucoraphanin.

### 2.5. Exogenous Hydrolysis of Glucoraphanin by Shewanella baltica Myr-37 Myrosinase

**Figure 6:**
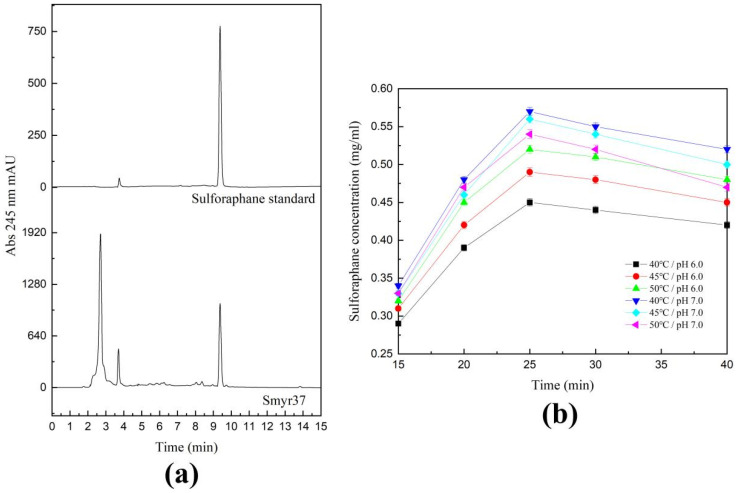
Optimization of the reactions for sulforaphane production by *Shewanella baltica* Myr-37 myrosinase. Due to the low purification efficiency, the crude enzyme of Smyr37 was used to hydrolyze glucoraphanin. The additive content of crude enzyme was 250 μg/mL. (**a**) High-performance liquid chromatography analysis at 245 nm of the content of sulforaphane in the hydrolysis reaction mixture. (**b**) Effects of temperature, pH, and reaction time on sulforaphane accumulation.

Sulforaphane is unstable, especially in alkaline environments. To explore the optimal reaction conditions for the production of sulforaphane by Smyr37, we carried out the hydrolysis of glucoraphanin at different temperatures (40 °C, 45 °C, and 50 °C) and pH values (6.0, 7.0). Then, we detected the sulforaphane content in the reaction solution using HPLC. As shown in Figure 6, when the reaction temperature was 40 °C and pH was 7.0, as high as 0.57 mg/mL sulforaphane could be detected within 25 min, and the corresponding conversion efficiency was 89%. However, with prolonged hydrolysis the sulforaphane yield decreased. When the hydrolysis time was more than 25 min, the sulforaphane content in an acidic environment (pH 6.0) decreased more slowly than that in a neutral environment (pH 7.0). These results indicated that Myr-37 myrosinase can be used to efficiently prepare sulforaphane from glucoraphanin.

## 3. Discussion

Myrosinase drives the health-promoting effects of glucosinolates via hydrolysis. Among the various hydrolysates, isothiocyanates have attracted much attention because of their potent anti-cancer activity. To improve the production of isothiocyanates, we need to mine more highly active myrosinases from nature. In this study, a bacterium Myr-37 produced myrosinase was isolated from the marine mud samples. The results of 16S rRNA sequencing indicated that this bacterium belonged to *S. baltica*. Furthermore, the characteristics of this novel myrosinase were evaluated and the hydrolysis conditions for the preparation of sulforaphane from glucoraphanin were optimized. 

Compared with studies on plant myrosinases, a limited number of studies have been conducted on bacterial myrosinases. Some gut microbes have been shown to be involved in glucosinolate metabolism and isothiocyanate production, indicating that these bacteria may be myrosinase producers [12]. However, to date, no gut microbes capable of producing myrosinase have been isolated. The first reported myrosinase Cmyr (66 kDa, sinigrin-induced enzyme) was purified from the *Citrobacter* strain WYE1, and the optimum temperature and pH for the myrosinase activity were 25 °C and 6.0, respectively [23]. *Leclercia adecarboxylata* myrosinases LAM3425 and LAM0641 are intracellular enzymes with a molecular weight of approximately 70 kDa. The optimum temperature and pH for their enzymatic activity were 25 °C and 6.6, respectively [24]. The *R. inusitata* myrosinase Rmyr (69 kDa) exhibited good glucoraphanin-hydrolysis activity, and the optimum temperature and pH for the myrosinase activity were 40 °C and 7.0, respectively [14]. 6-phospho-β-glucosidase from *E. coli* 0157:H7 and the BT2159-BT2156 operon from *Bacteroides thetaiotaomicron* have also been proved to be involved in the glucosinolate metabolism [12,26]. In the present study, a novel bacterial myrosinase, Smyr37 (100 kDa), was purified from the strain *S. baltica* Myr-37 (Figure 2 and Figure 4b). The optimum temperature and pH of Smyr37 were 50 °C and 8.0, respectively, which were significantly different from those of the four identified bacterial myrosinases (Figure 3a,b). The optimum temperature of Smyr37 was higher than that of the other bacterial myrosinases. However, this trait is similar to that of plant myrosinases, such as *Arabidopsis thaliana* myrosinase TGG1 and TGG2 (50 °C) and *Armoracia rusticana* myrosinase MYRII (50 °C) [27,28]. Smyr37, unlike most myrosinases, exhibited higher activity in neutral or slightly alkaline environments (Figure 3b). For instance, the optimal pH for TGG1, Cmyr, and ArMY2 are 5.5, 6.0, and 5.0, respectively [23,28,29]. As shown in Figure 3c, metal ions (1 mM), including Zn^2+^, Mg^2+^, Ag^+^, Fe^3+^, Cu^2+^, Ba^2+^, K^+^, Ca^2+^, Pd^2+^, and Co^2+^, inhibited Smyr37 activity, which was consistent with the characteristics of the plant myrosinase and bacterial myrosinase Rmyr [14]. These results indicated that Smyr37 is a novel myrosinase with certain enzymatic properties different from those of the identified myrosinases. 

Sinigrin is hydrolyzed by myrosinase to produce allyl-isothiocyanate, and it can also be desulfurized by sulfatase to form desulfo-sinigrin, which can be further hydrolyzed to glucose by β-glucosidases [30]. In this study, we prepared desulfo-sinigrin using sinigrin and sulfatase and analyzed the retention time of desulfo-sinigrin at 245 nm using HPLC (Figure 4b). However, within 60 min of the sinigrin hydrolysis reaction, desulfo-sinigrin was not detected, indicating that sinigrin degradation was not dependent on sulfatases. Furthermore, a single white band of BaSO_4_ appeared in the Native-PAGE gel (Figure 4c). That is, the white band (100 kDa) that appeared on the Native-PAGE gel was Smyr37. The molecular weight of Smyr37 observed in both SDS-PAGE gel and Native-PAGE gel was 100 kDa, indicating that Smyr37 might not be a dimer when it exerts myrosinase activity. Unlike Smyr37, many plant myrosinases are dimeric proteins [21,27,31]. As expected, Smyr37, similar to other bacterial myrosinases, showed a better affinity for sinigrin than glucoraphanin (Figure 5). However, the difference between the affinity of Smyr37 for glucosinolates and sulforaphane is minimal, indicating that the substrate-binding mechanism of Smyr37 to glucosinolates is worth investigating. In general, it is essential to analyze the genomic information of Smyr37, determine its structure, and further elucidate its reaction mechanism in future studies.

Isothiocyanates, the metabolites of glucosinolate hydrolyzed by myrosinase, have strong anti-cancer activity, with sulforaphane being the most effective active substance [32]. To better apply sulforaphane, many strategies, such as the polyethylene glycol ointment base, cyclodextrin inclusion complex, and microencapsulation embedding, have been used to improve the stability of sulforaphane [33]. In addition, the addition of exogenous myrosinase facilitates the conversion of glucoraphanin to sulforaphane [32]. At present, exogenous myrosinase is extracted and purified from the seeds of cruciferous plants, but the entire extraction process is complicated, resulting in a high cost of industrial application. Exploring the novel and efficient myrosinases is beneficial for improving the efficiency of isothiocyanates preparation. Under the optimal conditions (50 °C, pH 8.0), the highest enzyme activity of Smyr37 was 6.95 μmol/min/(mg protein). When sinigrin was used as the substrate, the bacterial myrosinase activities of Cmyr and Rmyr were 2.69 μmol/min/(mg protein) and 12.73 μmol/min/(mg protein), respectively [14,23]. However, the glucoraphanin hydrolytic activity of Smyr37 (5.87 μmol/min/mg protein) reached 84% of that of Rmyr (6.99 μmol/min/mg protein) [14]. Considering that sulforaphane is relatively stable in neutral and acidic environments, we optimized the conditions for sulforaphane preparation from glucoraphanin using Smyr37. The optimum pH of Smyr37 was 8.0, but sulforaphane accumulation was significantly affected in an alkaline environment. Under conditions of excess glucoraphanin, as high as 0.57 mg/mL of sulforaphane was detected within 25 min (Figure 6). When the first 25 min of the reaction was considered, the molar fractional conversion of the consumed glucoraphanin to sulforaphane was calculated to be approximately 89%, which was higher than that of many plant myrosinases [19,34]. However, the conversion efficiency of Smyr37 had some discrepancy with that of *R. inusitata* myrosinase Rmyr (97.8%) [14]. The lower conversion efficiency may be related to the preparation of sulforaphane using a crude enzyme. If the gene encoding of Smyr37 was heterologously expressed in the *E. coli* system, it was expected to obtain more pure enzymes, and the conversion efficiency of Smyr37 might be improved by using the pure enzyme.

Overall, results of molecular weight and catalytic properties demonstrate that Smyr37 is a novel myrosinase. Moreover, this is the first time that myrosinase has been reported in *S. baltica*, and the application of *S. baltica* Myr-37 or myrosinase Smyr37 needs to be further studied.

## 4. Materials and Methods

### 4.1. Chemical Reagents

Sinigrin (#3952-98-5), glucoraphanin (#21414-41-5), sulforaphane (#4478-93-7), and sulfatase (#9016-17-5) were obtained from Sigma-Aldrich (Shanghai, China). The genomic DNA extraction kit for gram-negative bacteria was purchased from Solarbio Life Sciences (Beijing, China). All other chemicals were purchased from Aladdin (Shanghai, China).

### 4.2. Screening of the Sinigrin-Degrading Strains from the Marine Mud

Marine mud samples collected at 119°34.228′ E, 34°45.076′ S, and a depth of 7.1 m in the Yellow Sea were used to isolate the myrosinase-producing strain, as previously described [24]. Briefly, 1 g of sample was added to 5 mL YPD liquid medium and cultured at 28 °C for 24 h under static conditions. Then, 100 μL of the culture was inoculated into 10 mL sterilized sinigrin M9 medium and incubated at 28 °C for 48 h. This process was repeated three times. Finally, the cultures were diluted tenfold with sterile saline and the diluent was spread onto a sinigrin M9 solid medium containing 2.5 mM BaCl_2_. Myrosinase can cleave sulfate bonds in glucosinolates, and SO_4_^2-^ reacts with Ba^2+^ to precipitate BaSO_4_. Therefore, a colony with precipitation may represent a sinigrin-degrading strain. We obtained over 65 colonies that could generate white precipitates on the sinigrin M9 plates containing Ba^2+^. The ability of the colony to degrade sinigrin was preliminarily analyzed using HPLC. The HPLC conditions are described in the following section.

### 4.3. Myrosinase Activity Assay

The hydrolysis of glucosinolates by myrosinase results in the production of glucose. Therefore, we evaluated the myrosinase activity by measuring the production of glucose, which was measured using the 3,5-dinitrosalicylic acid (DNS) colorimetric method. 

We measured the myrosinase activity of the 65 strains, as described below. Each colony was inoculated separately in LB medium and fermented at 28 °C for 24 h. Sinigrin solution (200 μL, 2 mg/mL) was added to 1 mL of fermentation broth or the supernatant of disrupted cells for estimating extracellular and intracellular myrosinase activity, respectively, and the mixture was incubated at 28 °C for 30 min. The absorbance of the reaction solution at OD540 was measured. The experiment was repeated three times. One unit (1 U) of myrosinase activity was defined as the amount of enzyme that releases 1 μmol of glucose per minute.

### 4.4. Identification of the Myrosinase-Producing Strain

The strain with the highest myrosinase activity among the 65 sinigrin-degrading strains was named Myr-37. Myr-37 was identified using the 16S rRNA gene sequence alignment and phylogenetic analysis. The 16S rRNA gene sequence, which was amplified from the genomic DNA of Myr-37 using the primers 27F and 1492R, was analyzed by Qingke Biotechnology (Qingdao, China). The sequencing data were submitted to NCBI GenBank and compared with public sequences in the EMBL database using BLAST. Phylogenetic analysis was performed using MEGA 7.0 (Mega Limited, Auckland, New Zealand).

### 4.5. Characterization of Myrosinase Smyr37 Produced by Shewanella baltica Myr-37

Due to the low abundance of Smyr37 in the fermentation broth, we used a crude enzyme to detect the enzymatic properties of Smyr37. We concentrated the fermentation broth using 10 kDa ultrafiltration tubes and replaced the broth with PBS (pH 7.0). We collected 500 mL of fermentation broth and obtained 10 mL of the crude enzyme dissolved in PBS (pH 7.0). 

PBS (pH 7.0) containing sinigrin (2 mg/mL) was used as the enzymatic reaction buffer for myrosinase. The crude enzyme and sinigrin (2 mg/mL) were incubated at different temperatures (20 °C, 30 °C, 40 °C, 50 °C, 60 °C, and 70 °C) for 30 min. Myrosinase activity was measured as previously described. Similarly, crude enzyme and sinigrin (2 mg/mL) were incubated at different pH values (4.0, 5.0, 6.0, 7.0, 7.5, 8.0, 9.0, and 10.0) for 30 min. An acetate buffer was used to achieve pH values of 4.0, 5.0, and 6.0. PBS was used to achieve the pH values of 6.0, 7.0, 7.5, and 8.0. Glycine-NaOH buffer was used to achieve the pH values of 8.0, 9.0, and 10.0. The different pH buffers mentioned above were used to prepare sinigrin solutions. The myrosinase activity was determined at the optimal temperature. 

To determine the effect of metal ions and EDTA on myrosinase activity, metal ions (Zn^2+^, Mg^2+^, Ag^+^, Fe^3+^, Cu^2+^, Ba^2+^, K^+^, Ca^2+^, Pd^2+^, Co^2+^) and EDTA were added to the enzyme reaction system, and the enzymatic activity of myrosinase was measured. The final concentrations of the metal ions and EDTA were 1 mM.

### 4.6. Native-PAGE

Sinigrin and BaCl_2_ were used to prepare Native-PAGE gels [31]. The native gel consisted of 3 mL 30% Acry/Bis, 1.875 mL 1 M Tris-HCl (pH 8.8), 0.45 mL 50% glycerol solution, 0.05 mL 10% APS solution, 0.005 mL TEMED, and 2.125 mL distilled water with sinigrin (2 mg/mL) and BaCl_2_ (50 mM). The fermentation supernatant (30 µL) was mixed with 7 µL of 5 × protein-loading buffer. Samples were then loaded onto a Native-PAGE gel with Ba^2+^. Electrophoresis was performed at 4 °C for approximately 1 h using a constant voltage (80 V) in 1 × Native-PAGE running buffer, and then continued for approximately 1 h at a constant voltage of 120 V. After electrophoresis, the Native-PAGE gel was soaked in PBS (pH 8.0) and kept at 37 °C for 2–6 h. Finally, we observed whether white precipitated bands similar to the protein bands appeared on the Native-PAGE gel.

### 4.7. Myrosinase Purification

The crude enzyme was used to purify Smyr37. The purification method resembles that of Albaser et al. [23]. The steps for myrosinase purification were as follows: A HiTrap Capto Q column was used for the primary separation of myrosinase, and the column was eluted with buffer A (20 mM Tris-HCl buffer containing 100 mM NaCl, pH 6.0) and buffer B (1 M NaCl) at a rate of 0.8 mL/min. The gradient profile was as follows: 0–50% buffer B (10 column volumes) and 50–100% buffer B (5 column volumes). The samples were pooled for myrosinase activity, and NaCl was removed by dialysis at 4 °C. The dialyzate with myrosinase activity was reloaded onto a HiTrap Capto Q column, and the column was eluted with buffer A (20 mM Tris-HCl buffer containing 100 mM NaCl, pH 6.0) and buffer B (1 M NaCl) at a rate of 0.75 mL/min. The gradient profile was as follows: 0–30% buffer B (3 column volumes), 30–50% buffer B (8 column volumes), and 50–100% buffer B (5 column volumes). SDS-PAGE analysis was used to estimate the purity of Smyr37. Then, the eluate containing the 100 kDa protein was collected. The collected fluid was further loaded onto a Superdex200 Increase 10/300 column for the third separation of myrosinase, and the column was eluted with buffer C (20 mM Tris-HCl buffer containing 200 mM NaCl, pH 6.0) at a rate of 0.5 mL/min. The myrosinase activity of each elution solution was determined after receiving the elution solutions with different protein peaks. 

### 4.8. HPLC Analysis 

Sinigrin, desulfosinigrin, glucoraphanin, and sulforaphane were analyzed and quantified using HPLC. Sinigrin and glucoraphanin solutions of different concentrations (0.2 mg/mL, 0.4 mg/mL, 0.6 mg/mL, 1.0 mg/mL, 1.5 mg/mL, and 1.5 mg/mL), were prepared. A standard curve was then constructed between the glucosinolate concentration and peak area. Desulfosinigrin was prepared as described by Tie et al. [24]. The HPLC analysis conditions were as follows: the analysis was performed on the Agilent 1260 system using a C18 column (ZORBAX SB-C18, 4.6 mm × 250 mm, 5 μm, Agilent Technologies, Santa Clara, CA, USA). Acetonitrile and water were used as the mobile phases. The flow rate and injection volume were 0.8 mL/min and 5 μL, respectively. The gradient profile was 0~8 min, 20~40% acetonitrile; 8~18 min, 40~60% acetonitrile; 18~20 min, 60~100% acetonitrile; 20~24 min, 100~20% acetonitrile; 24~25 min, 20% acetonitrile. 

Sulforaphane in the reaction solution catalyzed by Smyr37 was extracted and quantified according to the method described by Wang et al. [14]. The mobile gradient profile was 0~8 min, 20~40% acetonitrile; 8~20 min, 40~50% acetonitrile; 20~35 min, 50~60% acetonitrile; 35~43 min, 60~100% acetonitrile; 43~49 min, 100% acetonitrile; 49~50 min, 100~20% acetonitrile.

The detection wavelengths of sulforaphane and sinigrin (or desulfo-sinigrin or glucoraphanin) were 245 nm and 229 nm, respectively.

## 5. Conclusions

In summary, we isolated the bacterium *S. baltica* Myr-37, which is capable of producing a novel myrosinase, Smyr37, with a molecular weight of 100 kDa, from the marine mud samples in the Yellow Sea. Our results showed that Smyr37 could convert glucoraphanin to sulforaphane at a high level. Although the gene encoding Smyr37 in the genome of *S. baltica* Myr-37 is unknown, our data have demonstrated that Smyr37 is promising as an exogenous myrosinase to produce sulforaphane.

## Figures and Tables

**Table 1:** Purification of *Shewanella baltica* Myr-37 myrosinase.

Purification Step	Volume [mL]	Total Protein [mg]	Specific Activity [nmol/min/mg]	Yield [%]
Crude enzyme	10	107	662	100
Ion exchange first (HiTrap Capto Q column)	3	2.95	1245	5.18
Ion exchange second (HiTrap Capto Q column)	1.5	0.131	2297	0.42
Gelfiltration (Superdex200 Increase 10/300 column)	0.5	0.0159	6951	0.16

## Data Availability

The data presented in this study are available on request from the corresponding author.
